# Recurrent abdominal pain and upper gastrointestinal endoscopy findings in children and adolescents presenting at the Lagos University Teaching Hospital

**DOI:** 10.1371/journal.pone.0216394

**Published:** 2019-05-23

**Authors:** Oluwafunmilayo Funke Adeniyi, Emuobor Aghoghor Odeghe, Mary Adetola Lawal, Adebambo Olatunde Olowu, Adesoji Ademuyiwa

**Affiliations:** 1 Department of Paediatrics, University of Lagos/College of Medicine/ Lagos University Teaching Hospital, Lagos, Nigeria; 2 Department of Medicine, College of Medicine, University of Lagos / Lagos University Teaching Hospital, Lagos, Nigeria; 3 Department of Paediatrics, Lagos University Teaching Hospital, Lagos, Nigeria; 4 Department of Anaesthesia, Lagos University Teaching Hospital, Lagos, Nigeria; 5 Paediatric Surgery Unit, Department of Surgery, Lagos University Teaching Hospital, Idi Araba, Lagos, Nigeria; Osaka City University Graduate School of Medicine, JAPAN

## Abstract

**Introduction:**

Recurrent abdominal pain (RAP) is a common reason for referral to the paediatric gastroenterology unit and the attending physician needs to be able to rule out an organic cause when evaluating any child with this condition. The aim of this study was to describe the endoscopic findings in children who presented to the paediatric gastroenterology unit of the Lagos University Teaching Hospital (LUTH) with RAP.

**Methods:**

This was a prospective descriptive study which was conducted from January 2015 to July 2018 at the Paediatric Gastroenterology unit of the department of Paediatrics and the endoscopy unit of the LUTH, Lagos, Nigeria. All children and adolescents ≤ 19 years old with recurrent abdominal pain who were referred for upper GI endoscopy during the study period, were recruited. Baseline sociodemographic data, dyspepsia and alarm symptoms if present were documented. Results of other investigations namely stool examination for ova, parasites, occult blood and faecal antigen for Helicobacter pylori and abdominal scan were also documented.

**Results:**

A total of 113 children with recurrent abdominal pain was referred during the study period and 87 (76.7%) of them had upper GI endoscopy done. Out of the participants, 38(43.7%) were boys and 49(56.3) girls. Alarm features were present in 15(17.6%) and dyspepsia was seen in 22(25.3%) of the subjects. The main endoscopic findings were: gastritis in 39 (44.8%), gastric erosions in 14(16.2%), hiatus hernia in 7(8.1%), duodenitis in 6 (6.9%), gastric polyp in 4 (4.6%).

**Conclusion:**

Upper GI endoscopy remains an invaluable tool in the tool in evaluating RAP in children as it enables accurate diagnosis of GI causes of RAP. There is a need to advocate for easier access to this procedure in the developing countries.

## Introduction

Chronic or Recurrent abdominal pain (RAP) was first defined by Apley in 1958 as “three episodes of abdominal pain occurring in the space of three months, severe enough to affect daily activities”[1] Conventionally, RAP is believed to be a functional disorder and over the years the definition of RAP has been modified with the development of the Rome I to Rome III criteria[2] and more recently the Rome IV Criteria which now defines RAP as abdominal pain which occurs for ≥2 months with at least one episode of pain/week severe enough to affect children’s normal activity.[3,4] However, with the advent of new innovations and technological advancement, especially with diagnostic GI endoscopy, organic causes of RAP are increasingly been identified.[5]

RAP remains a common reason for referral to the general paediatrican and the paediatric gastroenterology unit in particular, and the attending physician needs to be able to rule out an organic cause when evaluating any child with this condition.[6] In order to identify organic causes of children with RAP the concept of the alarm features or “red flags” has been established[7] and it is believed that endoscopy is more frequently associated with abnormal findings in patients who have alarm features than in those without the red flag signs. Such alarm features include the presence of features such as weight loss, poor growth, GI bleeding, significant vomiting, abdominal tenderness, abdominal mass etc. [7]

In the Caucasian population, RAP has been well described with a prevalence of 10–20% in children in many reports. [8–11] However, in sub-Saharan Africa there is a dearth of literature on RAP and GI causes in children. The role of diagnostic upper GI endoscopy in unraveling such aetiologies has also been sparsely documented. Thus, the aim of this study is to describe the endoscopic findings in children with RAP who were seen at the LUTH, Nigeria in order to elucidate the organic causes of the condition.

## Methods

This was a prospective descriptive study which was conducted from January 2015 to July 2018 at the Paediatric Gastroenterology unit of the department of Paediatrics and the Endoscopy unit of the Lagos University Teaching Hospital, Lagos, Nigeria.

The inclusion criteria were children and adolescents ≤ 19 years old with recurrent abdominal pain who were referred for upper GI endoscopy, both from the outpatient clinics and inpatient wards during the study period and children and adolescents with no previous GI endoscopy procedures.

Exclusion criteria were: children with previous endoscopic diagnosis of GI disorder, use of PPIs within 2 weeks, critically ill children. Recurrent abdominal pain was defined as abdominal pain for ≥2 months with at least one episode of pain/week severe enough to affect children’s normal activity.

The data retrieved from the study participants were entered into a standard proforma. This included: complete history that focused on abdominal pain, dyspepsia and alarm features.

Dyspepsia was defined as the presence of troublesome pain or discomfort located in the upper part of the abdomen (epigastrium) and/or nausea, early satiety or uncomfortable feeling of fullness after meals, feeling of bloatiness after meals, nocturnal pain, heartburn, retrosternal pain, excessive belching and vomiting. [12]

The presence of alarm symptoms and signs associated with the abdominal pain namely GI bleeding, significant vomiting, weight loss, poor growth, chronic diarrhea, unexplained fever, persistent pain in the right upper quadrant or right lower quadrant, presence of abdominal mass, hepatomegaly, splenomegaly, perianal anomalies were also documented. Significant vomiting was defined as prolonged or protracted vomiting; bilious vomiting or a pattern of vomiting that was worrisome to the referring clinician. Other relevant symptoms were also documented in the standard proforma.

A thorough physical examination was also performed on all the study participants. Each child had a stool examination for ova, parasites, occult blood and antigen for Helicobacter pylori.

The stool fecal antigen test was carried with the use of the Rapid Strip HpSA ® manufactured by Meridian bioscience Europe S.r.L. This is a rapid 5 minutes immunoassay, based on a lateral flow chromatography technique that detects H. pylori antigens present in human stool by utilizing a monoclonal anti-H.pylori antibody. (See Appendix 1)

An abdominal scan was also carried out.

Other investigations carried out for each participant were based on the clinical suspicion of any other comorbid condition which may be present. Comorbid conditions were appropriately documented in the proforma.

### Endoscopic procedure

Upper GI endoscopy was performed for all the study participants following an overnight fast. Each procedure was done with the patient in the left lateral position with a Karlz Storz video endoscope (model 13821 PKS/NKS, Germany) and the diagnosis was based on visual assessment in collaboration with proficient endoscopists. A venous access was established for each of the study participant prior to the procedure in order to administer the drugs for sedation and the vital signs and oxygen saturation were monitored throughout the procedure. Each endoscopic procedure was carried out with the assistance of two endoscopy nurses.

### Sedation

Conscious sedation was performed for the study participants with the assistance of an experienced anaesthetist. Children below the age of 6 years were administered IV propofol at 1mg/kg, and IV Ketamine at 1mg/kg, with adequate monitoring of the vital signs. Oxygen saturation was also monitored with the pulse oximeter.

For children >6 years of age sedation was performed using 1% local lignocaine spray to anaesthetize the oropharynx. Subsequently, each child was given intravenous (IV) midazolam at 0.5 mg/kg, IV pentazocine at 0.5 mg/kg and IV hyoscine 0.5 mg/kg. None of the children had general anaesthesia, and there was no record of any adverse reaction to the sedative agents.

Histopathologic evaluation was also performed for the biopsies taken from some of the participants during the study. Gastritis was classified according to the updated Sydney system. [13]

### Statistical analysis

Data were entered and analysed using SPSS version 21 (SPSS Statistics for Windows, version 21.0, IBM Corp, USA). Basic descriptive statistics were performed and displayed as frequency tables.

### Ethical considerations

The study was approved by the Health Research and Ethics Committee of LUTH. The details of the procedure were explained to the parents prior to the procedure. Thereafter, a written informed consent was obtained from the parents, and an assent was also obtained from the child where appropriate, before he or she was enrolled in the study.

## Results

One hundred and thirteen (113) children and adolescents were referred to the paediatric gastroenterology unit on account of recurrent abdominal pain during the study period and 87 (76.9%) of them had Upper GI endoscopy done. The age range of the study participants was 1–19 years and the median age was 9 years. Table 1 shows the general characteristics of the study participants. Majority (60.9%) of the participants were in the 5–10 year age group. Thirty-eight (43.7%) of the participants were males and 49(56.3%) were females. M: F = 0.7: 1.

**Table 1:** General characteristics of the study participants.

Parameter	Frequency	Percentage(%)
**Age(years)**		
**<**5	10	11.5
5–10	53	60.9
>10	24	27.6
**Gender**		
Male	38	43.7
Female	49	56.3
**Religion**		
Christian	72	82.8
Muslim	15	17.2
**Ethnicity**		
Yoruba	53	60.9
Igbo	33	37.9
Hausa	1	1.1
**Presence of Alarm Symptoms**		
Haematemesis	4	4.6
Melena	2	2.3
Vomiting	3	3.4
Anaemia	1	1.1
Weight loss	1	1.1
**Presence of Dyspepsia**		
Yes	22	25.3
No	65	74.7
**Presence of Helicobacter pylori**		
Yes	31	35.6
No	56	64.3

Ethnicity-Tribe

A quarter (25.3%) of the children had dyspepsia. Alarm symptoms were present in 11(12.6%) of the participants. GI bleeding was present in 6.9% of the children, and was the most common alarm symptom. (Table 1) GI bleeding occurred secondary to the ingestion of NSAIDs and herbal preparations in 2 and 3 of the children respectively. One of the children had bleeding from esophageal varices.

Thirty one (35.6%) of the children were *H*.*pylori* positive using the monoclonal stool antigen test.

Table 2 shows the endoscopic findings in the study participants with RAP.

**Table 2:** The endoscopic diagnosis in the study participants with RAP.

Endoscopic Diagnosis	Frequency	Percent
Normal	11	12.6
Hiatus Hernia	7	8.1
*Esophagitis	4	4.6
**Portal Hypertensive gastropathy	1	1.1
Heterotrophic gastric mucosal patch	1	1.1
Gastritis	36	41.4
Gastric Polyp	4	4.6
Gastric ulcer	2	2.3
Gastric erosions	14	16.1
Duodenitis	6	6.9
Duodenal ulcer	1	1.1
Total	87	100

*3 of the children also had gastritis.

** This participant also had esophageal varices

In this study, 87.4% of the study participants who presented with abdominal pain had endoscopic abnormalities and 12.6% had normal findings. Gastritis (41.4%) was the most common endoscopic finding in the participants followed by gastric erosions and Hiatus hernia seen in 14 (16.1%) and 7(8%) of the study participants respectively. The least common endoscopic abnormalities were gastric polyps (4.6%), esophagitis (4.6%), gastric ulcers (2.3%) and duodenal ulcers (1.1%) see Table 2.

### Red flag/Alarm symptoms and endoscopic findings

Eleven (12.6%) of the study participants had red flag symptoms and 6 of these children with red flag signs had significant endoscopic findings giving an overall yield of 54.5%.

Six children had Upper GI bleeding (Hematemesis and Melena) and 4 of them had significant endoscopic findings namely gastritis and gastric erosions giving an endoscopic yield of 66.7%. Three (3) participants had significant vomiting and only 1 of them had an abnormal endoscopic finding (Hiatus Hernia) thus endoscopic yield was 33.3%. Anaemia was seen in 1 child and Gastritis was seen on endoscopy. Weight loss was also present in 1 participant however the endoscopy was normal in this instance.

### Dyspepsia and endoscopic findings

Twenty-two (22) study participants presented with dyspepsia and 20(90.9%) of these participants had significant endoscopic findings. Eleven (50%) of the dyspeptic children had gastritis, while 2(9.1%) had gastric erosions. Each of the remaining 7 participants had Hiatus hernia (4.5%), esophagitis (4.5%), heterotrophic gastric patch (4.5%), gastric ulcer (4.5%), gastric polyp(4.5%), duodenitis (4.5%) and duodenal ulcer (4.5%) respectively.

### Histopathology findings

Twenty-six (29.8%) of the study participants had biopsies taken and sent for histopathologic evaluation.

The polyps seen in the study were inflammatory polyps in 4 of the children. Only 8 of the children with erosions had biopsies and histology done and the erosions were confirmed histologically. Eleven of the children with endoscopic gastritis (7 with antral gastritis and 4 with nodular gastritis) also had biopsies and histology done. See Table 3

**Table 3:** Histopathology findings in the study participants.

Endoscopic findings	Histology findings
Polyps(4)	Inflammatory Polyp
Gastric Erosions(8)	Foci of superficial ectatic blood vessels were also seen
Antral gastritis(7)	4 of the children with antral gastritis had mild to moderate chronic gastritis 3 children had chronic atrophic gastritis
Nodular gastritis (4)	2 of the participants with nodular gastritis were H.pylori positive
Duodenitis (2)	Histology showed mild to moderate inflammation

Gastritis a form of gastropathy refers to inflammation of the stomach that may occur from an infection or auto-immunological process.[14] Gastropathy on the other hand refers to pathology of the stomach with evident epithelial damage and regeneration which may be due to be intrinsic or extrinsic irritants.[14] Gastropathy was a prominent feature in this cohort of study participants and the types of gastropathy seen in the study participants are highlighted in Table 4. Gastric erythema was seen in more than a third of the children, and the least prevalent gastropathy was biliary gastritis.

**Table 4:** Gastropathies seen in the study participants.

Type of gastropathy	Number (N = 53)	Percentage (%)
Gastric erythema	19	35.8
Gastric erosions/Erosive Gastritis	14	26.4
Antral gastritis	10	18.9
Nodular/Follicular gastritis	8	15.1
Portal Hypertensive gastropathy	1	1.9
Biliary gastritis	1	1.9

The associated comorbidities observed in the study participants are shown in Table 5.

**Table 5:** Associated comorbidities in the study participants with RAP.

Comorbodity	Frequency	Percentage
None	80	92.3
Sickle cell disease	2	2.2
Multicystic dysplastic kidney	1	1.1
Food allergy	1	1.1
CP	1	1.1
Benign Intracranial Hypertension	1	1.1
Wilms tumor	1	1.1

CP-Cerebral Palsy, RAP-Recurrent Abdominal pain

Two of them had sickle cell disease, while 5 of the children had other comorbid conditions as outlined in Table 4.

## Discussion

RAP remains a condition with significant public health importance, though previously most cases had been designated as functional. However, with the advent of GI endoscopy the finding of organic causes of RAP in children has been on the increase. In this study, 87 gastroscopies were performed for children and adolescents who presented with the symptom in order to elucidate possible underlying GI causes.

Endoscopic abnormalities were seen in 76 (87.4%) of the children. This finding is similar to that of Motamed et al [15] in Iran who observed that 84% of children with RAP had organic endoscopic abnormalities. However, in the latter study gastroscopy was performed only for children with alarm symptoms or red flag signs and this may explain the high figure observed. In our centre, not all children with RAP undergo gastroscopy as children are only referred for the procedure when other investigations have not unraveled an underlying cause and the referring doctor still has a strong suspicion for an underlying organic pathology. This may possibly explain the high prevalence of endoscopic abnormalities seen in our study. Nevertheless, recent reports from Ivory Coast by Banguorou et al [16] and Upadhyay et al [17]in Nepal where gastroscopies were performed for all patients who presented with RAP observed endoscopic abnormalities in up to 70% and 71% of the children respectively, while other workers have reported lower figures in children with RAP without red flag signs, as observed by Aanpeureng et al (51.6%)[18] and Urakapol et al (44.7%).[5] Thus, it appears that the endoscopic yield for children without alarm symptoms is variable, with documented figures between 38–58% [5,19]

Nowadays, endoscopy is advocated particularly for children with red flags or alarm symptoms and this may be a practical approach to evaluating children with RAP [19]. The physician’s clinician acumen, however, plays a huge role in determining who should be referred for further endoscopic evaluation. In our study, 87 of the 113 patients referred for gastroscopy actually had gastroscopy done, but only 12.6% reported alarm symptoms. Thus, there is still a need to ascertain the sensitivity and predictive value of this concept in the African children in different parts of the region.

The most common endoscopic findings in this current study were gastropathies, particularly gastritis. This is similar to the findings by Bangoura et al in Ivory coast [16] and Upadhyay et al [17]in Nepal, and in other developing countries.[20,21]The high rate of gastritis in our study may be linked to the high prevalence of H.pylori infection in Nigeria, and in our cohort more than a third (35.6%) of the participants were H.pylori positive. The participants in this study were screened for H.pylori with the use of the monoclonal fecal antigen test. The gold standard for H.pylori diagnosis/evaluation is the use of rapid urease testing, histology and urea breath tests (13)C-UBT).[22,23] The UBT have been found to have a high sensitivity(95.0%), specificity(98.4%) and accuracy (96.4%)but is not readily available in our setting and difficult to use in the younger children. However, the use of the monoclonal test is less expensive and has also been found to be an appropriate alternative to the UBT as studies have also documented similar sensitivity (98.3%), specificity (98.4%) and accuracy (98.3%) values for this test compared to the UBT. [24]

However, few of the participants in our study had gastric or duodenal ulcers. This finding is similar to reports from Iran [15] and Scandinavia [25].In Uganda (14.8%) [20] and Kathamandu (13%) [21] higher rates of ulcers were documented. Nevertheless, the endoscopic findings of gastritis and ulcers have been found to manifest clinically with epigastric pain as was observed in this present study.

Lower rates of gastropathies have been documented in the industrialized countries and in contrast to the findings in our study, endoscopic reports of children with RAP from countries in the Middle East (Iran) have documented high rates of esophagitis and hiatus hernia in the cohorts with abdominal pain.[15,16] Thus, it appears that there may be some geographical or climatic variation which may be related to genetics or differences in social class that play a role in the endoscopic findings in different regions of the world.[16]

Amongst the gastropathies observed gastric erythema was the most common type seen in this present study, similar to findings in Ivory Coast, Uganda and Nepal. [15,20,17] Gastric erosions were also a prominent finding in our study This may occur secondary to the ingestion of Non-steroidal anti-inflammatory agents and herbal preparations by some of the children was the only positive trigger that could explain the finding in these children. The administration of these agents to children is a common practice by many parents in south western Nigeria, and this may have contributed to the finding in our study. Antral gastritis and Nodular gastritis which are typically seen in H.pylori infection were seen in 18.9% and 15.1% of our study participants. The reason for this finding may be a reflection of the previous use of antibiotics and PPIs which is used empirically to treat abdominal pain in many parts of the country.

Bile gastritis which was seen in only one participant in our study is not a very common phenomenon. This has been documented by Bangorou et al in Ivory Coast and was attributed to vomiting efforts during the procedure which also observed in our study. [16]

Majority of children with dyspepsia (90.9%) in the present study had significant endoscopic abnormalities. Similar reports have been made by other authors [20, 21] while conflicting reports has been documented by other workers. [16–18] Nevertheless, endoscopy still plays a beneficial role in the evaluation of dyspepsia in the paediatric age group with dyspepsia.

Seven participants in the study had comorbid conditions but the relationship between these conditions and RAP is difficult to ascertain. However, it is known that sickle cell disease patients often complain of RAP which is usually secondary to vasoocclusive crises. The child with benign intracranial hypertension was on Diamox and few reports have documented that this drug may induce gastritis.[26] Food allergy may also cause abdominal pain when an offending allergen is consumed. However, the fact remains that functional pain should be least considered in patients with other comorbid conditions and it is mandatory that an underlying organic pathology be ruled out before concluding that the abdominal pain in that instance is functional.

One of the limitations in this study was that biopsy for histopathological evaluation was done in very few of the children and this was due to logistic and financial challenges.

In conclusion, upper GI endoscopy plays a significant role in unraveling the cause of RAP in children, especially in the presence of alarm features. The procedure has proven to be an effective diagnostic tool which is now been used to evaluate children of all ages. Endoscopy enables direct visualization of the GI tract and when combined with histopathologic evaluation gives accurate diagnosis and also enables therapeutic interventions. There is a need for further studies to ascertain the sensitivity of the alarm features in African children. Screening for H.pylori is advocated for children who present with RAP in developing countries.

## Supporting information

S1 File Appendix1: Rapid strip helicobacter pylori stool antigen test. (DOCX)

S1 Database Data set on abdominal pain and endoscopy. (SAV)
