# Supplementary material for: Recurrent abdominal pain and upper gastrointestinal endoscopy findings in children and adolescents presenting at the Lagos University Teaching Hospital
Source: PLoS One. 2019 May 23;14(5):e0216394. doi: 10.1371/journal.pone.0216394 (PMC6532862; doi:10.1371/journal.pone.0216394)
Supplement: S1 File — (DOCX) [file pone.0216394.s001.docx]

**Appendix 1**

**Rapid Strip Helicobacter Pylori Stool Antigen Test**

The Rapid Strip *H. pylori* Stool Antigen (HPSA) (Meridian Bioscience Europe ) is an *in vitro* qualitative test for the detection of *H. pylori* antigens in human stool. It is a rapid 5 minutes immunoassay, based on a lateral flow chromatography technique that detects *H. pylori* antigens present in human stool by utilizing a strip which contains monoclonal anti-*H. pylori* antibody. The strip was introduced in a test tube containing diluted patient samples and the appearance of a pink-red line in the reading area indicates a positive result after 5 minutes of incubation at room temperature. The procedure was carried out according to the manufacturer’s instruction as follows

1. One ml of sample diluent was transferred into a test tube.

2. A sample portion of approximately 5-6mm size is added with a wooden applicator and shaken gently in order to suspend it into the diluent.

3. It was vortexed for 15 seconds.

4. After 3 minutes when the solid particles had settled, 500 microliters of the supernatant was transferred into another test tube, with a pipette.

5. The reaction strip was dipped into the second test tube with the arrow pointing to the bottom. The liquid was not allowed to reach the blue area above the arrowheads.

6. The results were read after exactly 5 minutes in the white area. (Figure I)

For procedural control, a coloured line will always appear in the control line region, indicating that proper volume of specimen has been added and membrane wicking has occurred. However the manufacturer’s instruction was adhered to strictly because humidity and temperature can adversely affect the results. The sensitivity of the test is reduced when Proton Pump Inhibitors, bismuth or antibiotic is used two weeks prior to the

**INTERPRETATION OF RESULTS**

**Negative test result**: Only one BLUE coloured band (Control Line) appears across the white central area of the reaction strip. *H. pylori* antigens are absent or below the level of detection.

**Positive test result**: In addition to the BLUE band, a distinguishable PINK-RED band (Test Line) also appeared across the white central zone of the reaction strip. The intensity of the band was variable depending on the antigen concentration in the specimen. Any pink-red line, even very weak, was considered as a positive result. Any line or colour appearing after 5 minutes was of no diagnostic value. A positive test line indicates that there are detectable *H. pylori* antigens in the specimen.

**Invalid test result**: the BLUE band (Control Line) is absent, with or without a visually detectable PINK-RED band (Test Line). (Figure II)

The test tube was capped, vortexed for 15 seconds and allowed to wait for 3 minutes

Wooden applicator stick was used to collect sample and suspend in diluent.

Test tube containing 1ml of diluent.

A micropipette was used to transfer 500 microliter of supernatant to another test tube

The Rapid strip HpSA was dipped into the supernatant and read after 5 minutes.

Figure I: HPSA Test Procedure

Figure II: HPSA Interpretation of results.
